# Reduction of false positives using zone-specific prostate-specific antigen density for prostate MRI-based biopsy decision strategies

**DOI:** 10.1007/s00330-024-10700-z

**Published:** 2024-03-28

**Authors:** Charlie A. Hamm, Georg L. Baumgärtner, Anwar R. Padhani, Konrad P. Froböse, Franziska Dräger, Nick L. Beetz, Lynn J. Savic, Helena Posch, Julian Lenk, Simon Schallenberg, Andreas Maxeiner, Hannes Cash, Karsten Günzel, Bernd Hamm, Patrick Asbach, Tobias Penzkofer

**Affiliations:** 1 Department of Radiology, Charité – Universitätsmedizin Berlin, Corporate Member of Freie Universität Berlin, Humboldt-Universität zu Berlin, and Berlin Institute of Health, Berlin, Germany; 2 Berlin Institute of Health (BIH), Berlin, Germany; 3 Paul Strickland Scanner Centre, Mount Vernon Hospital, Northwood, Middlesex UK; 4 Institute of Pathology, Charité – Universitätsmedizin Berlin, Corporate Member of Freie Universität Berlin, Humboldt-Universität zu Berlin, and Berlin Institute of Health, Berlin, Germany; 5 Department of Urology, Charité – Universitätsmedizin Berlin, Corporate Member of Freie Universität Berlin, Humboldt-Universität zu Berlin, and Berlin Institute of Health, Berlin, Germany; 6 Department of Urology, Otto-von-Guericke-University Magdeburg, Germany and PROURO, Berlin, Germany; 7 Department of Urology, Vivantes Klinikum Am Urban, Berlin, Germany

**Keywords:** Prostatic neoplasms, Magnetic resonance imaging, Prostate-specific antigen density, Clinical decision-making, Image-guided biopsy

## Abstract

**Objectives:**

To develop and test zone-specific prostate-specific antigen density (sPSAD) combined with PI-RADS to guide prostate biopsy decision strategies (BDS).

**Methods:**

This retrospective study included consecutive patients, who underwent prostate MRI and biopsy (01/2012–10/2018). The whole gland and transition zone (TZ) were segmented at MRI using a retrained deep learning system (DLS; nnU-Net) to calculate PSAD and sPSAD, respectively. Additionally, sPSAD and PI-RADS were combined in a BDS, and diagnostic performances to detect Grade Group ≥ 2 (GG ≥ 2) prostate cancer were compared. Patient-based cancer detection using sPSAD was assessed by bootstrapping with 1000 repetitions and reported as area under the curve (AUC). Clinical utility of the BDS was tested in the hold-out test set using decision curve analysis. Statistics included nonparametric DeLong test for AUCs and Fisher-Yates test for remaining performance metrics.

**Results:**

A total of 1604 patients aged 67 (interquartile range, 61–73) with 48% GG ≥ 2 prevalence (774/1604) were evaluated. By employing DLS-based prostate and TZ volumes (DICE coefficients of 0.89 (95% confidence interval, 0.80–0.97) and 0.84 (0.70–0.99)), GG ≥ 2 detection using PSAD was inferior to sPSAD (AUC, 0.71 (0.68–0.74)/0.73 (0.70–0.76); *p* < 0.001). Combining PI-RADS with sPSAD, GG ≥ 2 detection specificity doubled from 18% (10–20%) to 43% (30–44%; *p* < 0.001) with similar sensitivity (93% (89–96%)/97% (94–99%); *p* = 0.052), when biopsies were taken in PI-RADS 4-5 and 3 only if sPSAD was ≥ 0.42 ng/mL/cc as compared to all PI-RADS 3-5 cases. Additionally, using the sPSAD-based BDS, false positives were reduced by 25% (123 (104–142)/165 (146–185); *p* < 0.001).

**Conclusion:**

Using sPSAD to guide biopsy decisions in PI-RADS 3 lesions can reduce false positives at MRI while maintaining high sensitivity for GG ≥ 2 cancers.

**Clinical relevance statement:**

Transition zone-specific prostate-specific antigen density can improve the accuracy of prostate cancer detection compared to MRI assessments alone, by lowering false-positive cases without significantly missing men with ISUP GG ≥ 2 cancers.

**Key Points:**

• *Prostate biopsy decision strategies using PI-RADS at MRI are limited by a substantial proportion of false positives, not yielding grade group ≥ 2 prostate cancer.*

• *PI-RADS combined with transition zone (TZ)-specific prostate-specific antigen density (PSAD) decreased the number of unproductive biopsies by 25% compared to PI-RADS only.*

• *TZ-specific PSAD also improved the specificity of MRI-directed biopsies by 9% compared to the whole gland PSAD, while showing identical sensitivity.*

**Supplementary Information:**

The online version contains supplementary material available at 10.1007/s00330-024-10700-z.

## Introduction

The introduction of the MRI pathway for the detection of clinically significant prostate cancer (csPCa) has reduced the need for prostate biopsy and lowered the detection of clinically insignificant cancers [1, 2]. The MRI pathway has also increased the accuracy of biopsies through improved targeting and grading with the majority of studies showing non-inferiority of csPCa detection [1–4]. Employing the Prostate Imaging-Reporting and Data System (PI-RADS) before biopsy still encounters a relatively high proportion of false positives in men considered to have csPCa with high inter-center variability, especially in men with PI-RADS 3 scoring [2, 5–7]. Thus, there is an ongoing clinical need for additional markers to reduce the number of false-positive cases to enable a more accurate triage of patients for biopsy.

Recent studies have addressed this unmet clinical need by using prostate volume (PV) normalized prostate-specific antigen (PSA density; PSAD) for biopsy management decisions [8–10]. Several studies have shown that PSAD and prostate MRI suspicion score (e.g., PI-RADS) are independent predictors of csPCa at biopsy [11–13]. In this context, PSAD values greater than 0.15 ng/mL/cc are more indicative of the presence of csPCa, whereas values less than 0.09 ng/mL/cc largely exclude csPCa [4, 14–16]. Furthermore, biopsy decision strategies (BDS), a combination of the PI-RADS scoring and PSAD, have been developed to maximize the accuracy of image-derived information for biopsy planning [14, 15, 17].

The PV is needed for PSAD to be calculated. However, the lack of standardized recommendations for PV determination limits the clinical utility of PSAD, as PV estimates do vary by imaging modality and calculation approach. Both planimetry and the prolate ellipse formula are used clinically [18–21]. Transition zone (TZ)-specific (s)PSAD has been postulated as being a more relevant test because the secretory cells of the TZ are primarily responsible for PSA production [22, 23]. TZ volume due to benign prostatic hyperplasia would better account for PSA values for biopsy decisions [24]. However, there is no specific formula for calculating TZ volume, which poses a barrier to the application of sPSAD in routine clinical practice.

In this context, deep learning (DL) systems have shown greater accuracy for whole prostate and TZ segmentation tasks [25–29]. However, to our knowledge, the diagnostic utility of DL-based automated sPSAD calculation for the detection of csPCa has not been comprehensively explored.

Therefore, the aim of this study was to develop and test a decision strategy combining DL-based sPSAD and PI-RADS to guide prostate biopsies in men at elevated risk of having PCa.

## Methods

This study complies with the Checklist for Artificial Intelligence in Medical Imaging and the Standards for Reporting Diagnostic Accuracy studies. The study was approved by the institutional review board and informed consent was waived due to its retrospective design.

### Study sample

We included consecutive men undergoing prostate biopsy at our institution between January 2012 and October 2018 as well as transrectal ultrasound (TRUS) and prostate MRI within 6 months before biopsy. Age, serum PSA, and TRUS-based PV were obtained from clinical records. Eligible MRI studies were retrieved from the institution’s PACS and incorporated into a customized DICOM-viewer for image annotation, quality assessment, and post-processing. A subset of this cohort was included in previous analyses, where PSAD was not investigated for PCa detection [30].

**Fig. 1:**
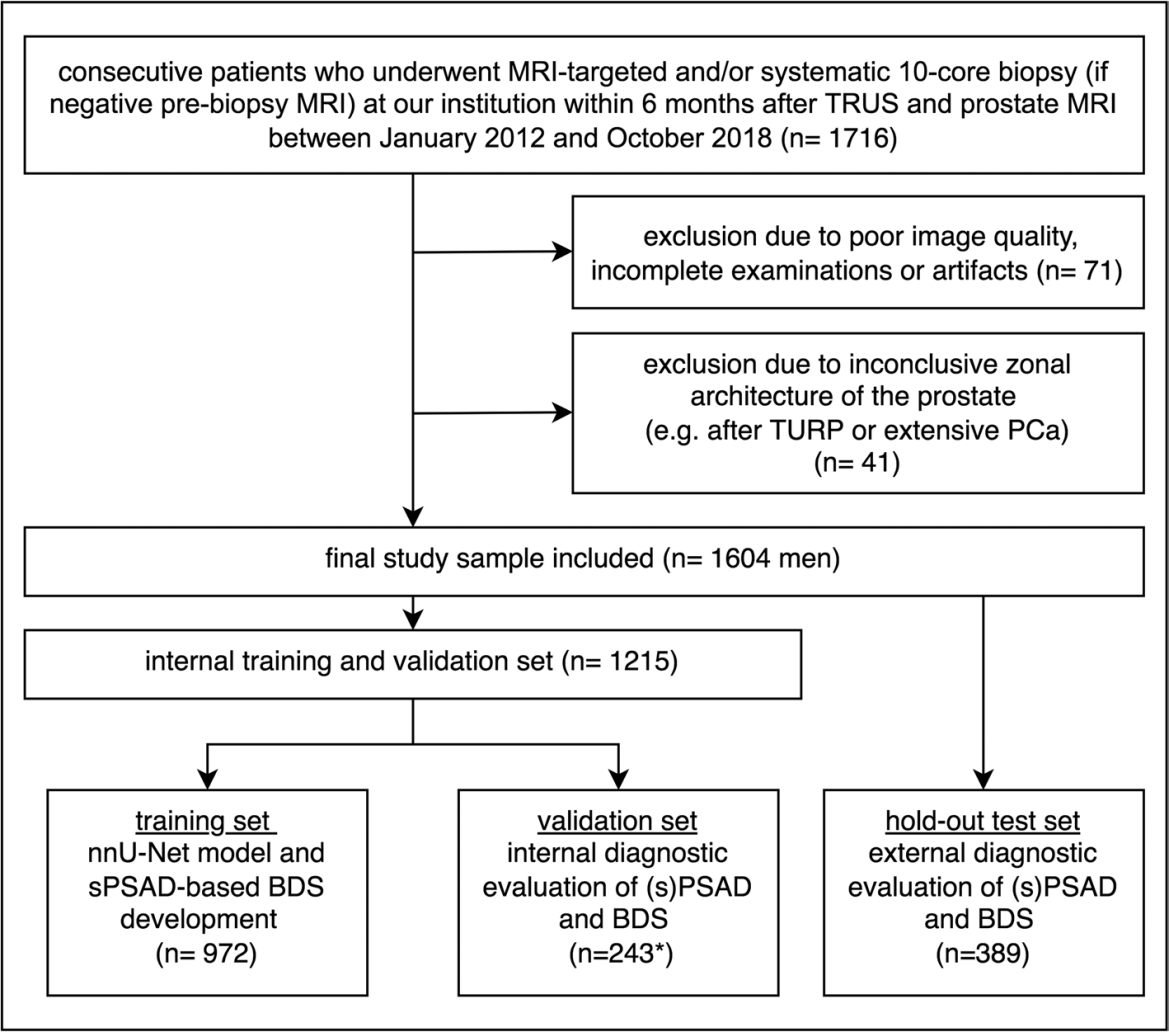
Patient flow diagram of the study sample. Fifteen men were missing a Prostate Imaging-Reporting and Data System (PI-RADS) score in the validation set (*). Thus, biopsy decision strategies (BDS) using prostate-specific antigen density (PSAD) or transition zone-specific (s)PSAD and PI-RADS were assessed in a subset of 228 men, while diagnostic performance of PSAD and sPSAD alone was assessed using the entire validation set (*n* = 243). A hold-out test set (*n* = 389) was used for testing the different BDS combining transition zone- or whole gland-based PSADs and PI-RADS scoring (*TRUS* transrectal ultrasound; *TURP* transurethral resection; *PCa* prostate cancer)

Patients with discrepancies in the radiologic-pathologic correlation, inconclusive zonal architecture of the prostate (e.g., indistinct or distorted zonal margins after transurethral resection), MRI using an endorectal coil, and MRI of insufficient quality were excluded (Fig. 1). Finally, the study sample was split into a training (61%), validation (15%), and hold-out test set (24%) comprising consecutive cases from 01/2012 to 01/2016, 01/2016–01/2017, and 01/2017–10/2018, respectively. For external testing, the publicly available prostate-MRI-US-biopsy set was used with a reported csPCa prevalence of 58% (399 of 692); the data set is accessible at https://wiki.cancerimagingarchive.net and detailed in the supplementary material [31].

### MRI protocol and PI-RADS scoring

MRI was acquired according to the PI-RADS and ESUR technical recommendation guidelines [5, 32]. Imaging was performed on three MRI scanners, including a 1.5-T (Magnetom Avanto, Siemens Healthineers) and two identical 3-T MRI scanners (Magnetom Skyra, Siemens Healthineers). Prostate MRI protocols comprised T2-weighted imaging (T2W), diffusion-weighted images (DWI), and a dynamic-contrast enhanced (DCE) sequence, if available. Scanning parameters were axial and coronal T2W images (3.0 × 0.47 × 0.47 mm, 18 cm FoV), axial DWI at *b* values of 0, 50, 500, and 1000 s/mm^2^ with mono-exponentially calculated apparent diffusion coefficient (ADC) maps, and extrapolated high-*b*-value DWI images (3.0 × 1.4 × 1.4 mm, 22 cm FoV, calculated *b* = 1400 s/mm^2^). All MR images were reviewed for sufficient image quality according to national diagnostic requirements for prostate MRI prior to analysis [33]. Under the supervision of expert radiologists (P.A., B.H., and T.P. each with > 10 years of experience), prostate lesions described by radiology faculty on primary reports were reviewed in consensus by two radiologists (C.H. and N.B. with 3–5 years of experience) using the PI-RADS v.2.1 criteria [5]. Up to four lesions per patient were identified, of which the lesion with the highest reported PI-RADS score was defined as the index lesion for the patient-based analysis. According to PI-RADS v.2.1 guidelines, PV was calculated using the prolate ellipsoid formula: width × height × length × π/6.

### TRUS-based volumetry and biopsy procedures

All TRUS examinations and MRI/US fusion-guided targeted biopsies were performed by a team of urologists (H.C., A.M., and K.G. each with > 10 years of experience) at our tertiary university center and in accordance with the European Association of Urology (EAU; 2022) guidelines. According to standard clinical protocol, PV was calculated using the prolate ellipsoid formula.

First, a targeted biopsy of the prostate with three cores per target lesion was performed as described previously [34]. Secondly, a systemic biopsy was performed with 10 cores from the apex, lateral mid-gland, base, and ventral and paraurethral region bilaterally, respectively. If pre-biopsy MRI was negative (PI-RADS 1-2), only systematic biopsy was performed. All cores were potted and documented separately, and examined and analyzed by experienced pathologists (including S.S.) following the ISUP guidelines [4].

### Retraining of a deep learning system for prostate segmentation at MRI

#### Data set and manual prostate segmentation

In the training and validation set, the whole gland (WG) and TZ were manually segmented by two investigators (K.F. & F.D.) under the supervision of two radiologists (C.H. and T.P. with 3 and > 10 years of experience, respectively). Segmentations were performed using the Medical Imaging Interaction Toolkit (MITK; v.2021.10, German Cancer Research Center, Division of Medical Image Computing) and 3D Slicer (v.4.10.2) [35]. Segmentation was performed according to the anatomical landmarks on axial and coronal T2W volumes, specified in the PI-RADS guidelines [5].

#### DL segmentation model

For automated segmentation of the prostate and TZ, a high-performance, well-established nnU-Net was selected and trained on T2W, ADC, and DWI axial images and the manually generated WG and TZ three-dimensional masks [36]. The training and validation set comprised 972 and 243 men, respectively [36]. The model architecture and training is detailed in the supplementary material. Segmentation performance of the DL system was assessed in the validation set calculating the DICE coefficient ranging from 0 to 1 (0 indicating no spatial overlap of binary segmentations; 1 indicating complete overlap). Moreover, a more comprehensive pairwise agreement test of PVs derived from different MRI- and TRUS-based segmentation approaches was performed separately as detailed in the supplementary material.

### Development of a biopsy decision strategy using PI-RADS and sPSAD

For BDS development using PI-RADS and sPSAD, the optimal sPSAD cutoff value was derived from the training set by selecting the cutoff value that achieved the highest specificity without suffering a statistically significant loss in sensitivity in csPCa detection. This was done by (I) calculating sPSAD specificity in incremental steps of 5% from 0 to 100%, (II) measuring the diagnostic performance of the BDS performing biopsy in men with PI-RADS 4-5 or PI-RADS 3 and sPSAD ≥ cutoff value using bootstrapping with 1000 repetitions, and (III) selecting the optimal sPSAD cutoff value using the exact Fisher-Yates test (alpha = 0.05).

### Outcomes and statistics

The primary outcome of this study was the detection rate of csPCa (Gleason Score ≥ 3 + 4; ISUP Grade Group (GG) ≥ 2) using sPSAD alone or in combination with PI-RADS scoring.

Patient-based detection accuracy of GG ≥ 2 in the validation set using TRUS- and MRI-based PSAD and sPSAD, respectively, was reported using bootstrapping over 1000 repetitions, and areas under the receiver operating characteristic (ROC) curve (AUCs) were plotted. AUCs were compared using the nonparametric approach proposed by DeLong et al [37].

GG ≥ 2 detection accuracy of the BDS using PI-RADS and sPSAD was evaluated in the validation and the hold-out test set calculating specificity and sensitivity. Finally, the potential added clinical value of BDS for GG ≥ 2 detection was compared with the PI-RADS 3-5 category alone using decision curve analysis (DCA) and the exact Fisher-Yates test. One-sided *p*-values < 0.05 were considered statistically significant. Statistical analyses were performed using Python (v.3.8.10) with the libraries scipy (v.1.5.4) and Scikit-learn (v.0.23.2).

## Results

### Study population

This study included 1604 patients, which were split into a training (*n* = 972), validation (*n* = 243), and hold-out test set (*n* = 389; Table 1). Men in this study were 67 (IQR, 61–73) years of age, had PSA of 7.85 (4.86–10.84) ng/mL, and were diagnosed with GG ≥ 2 PCa in 48% (774/1604). GG ≥ 2 disease was suspected at MRI in 1059 men (524 and 535 with PI-RADS 4 and 5, respectively), while 182 men had a PI-RADS category of 3. Seventy-one and 41 patients were excluded due to insufficient image quality and inconclusive zonal architecture of the prostate, respectively.

**Table 1:** Patient, histopathological, and PI-RADS characteristics

Characteristics	All men (*n* = 1604)	Training set (*n* = 972)	Validation set (*n* = 243)	Hold-out test set (*n* = 389)
Age (y; median, IQR)	67 (61–73)	66 (61–72)	67 (61–74)	66 (60–72)
PSA level (ng/mL; median, IQR)	7.85 (4.86–10.84)	8.55 (5.15–11.95)	7.82 (5.32–10.32)	6.31 (4.17–8.45)
Number of biopsy-naïve patients (%; *n*)	48% (763)	32% (313)	51% (12 5)	84% (325)
Number of patients with PCa (%; *n*)	69% (1109)	65% (632)	78% (189)	74% (288)
Number of patients with ISUP GG ≥ 2 cancers (%; *n*)	48% (766)	46% (449)	51% (123)	50% (194)
ISUP Grade (%; *n*)				
1	21% (343)	19% (183)	27% (66)	33% (94)
2	18% (289)	15% (149)	20% (48)	32% (92)
3	8% (123)	7% (71)	8% (20)	11% (32)
4	16% (264)	18% (172)	18% (43)	17% (49)
5	6% (90)	6% (57)	5% (12)	7% (21)
Highest patient-based PI-RADS (%; *n*)				
1/2	16% (260)	20% (198)	11% (26)	9% (36)
3	11% (182)	10% (102)	9% (21)	15% (58)
4	33% (524)	25% (243)	37% (89)	49% (191)
5	33% (535)	35% (338)	38% (92)	27% (104)
NA	6% (103)	9% (91)	6% (15)	-

*PSA* prostate-specific antigen, *PCa* prostate cancer, *ISUP GG* International Society of Urological Pathology Grade Group, *PI-RADS* Prostate Imaging-Reporting and Data System

### Prostate volume and PSAD

Across the whole study sample prostate volumetry on Segmentation-MRI, Ellipsoid-MRI and Ellipsoid-TRUS revealed a WG volume of 50 (95% confidence interval (95%CI), 22–78) mL, 55 (22–88) mL, and 55 (26–84) mL (*p* < 0.001, Table 2) with a PSAD of 0.26 (0–0.65) ng/mL/cc, 0.25 (0–0.56) ng/mL/cc, and 0.24 (0–0.63) ng/mL/cc (*p* < 0.001), respectively. The Ellipsoid-based approach at MRI and TRUS overestimated PV by 10% (55 vs 50 mL; *p* < 0.001, Supplementary Figure 1). The average MRI-based TZ volume was 31.26 mL and resulted in a sPSAD of 0.55 (0–1.41) ng/mL/cc.

**Table 2:** MRI and TRUS volumetry of the prostate and calculated PSA density

Characteristics	All men (*n* = 1604)	Training set (*n* = 972)	Validation set (*n* = 243)	Hold-out test set (*n* = 389)
Prostate volume (mL) mean ± SD, range				
Whole gland TRUS-based	55 ± 29 (6–350)	57 ± 29 (6–190)	57 ± 26 (15–180)	50 ± 31 (16–350)
Whole gland MRI-based	50 ± 28 (10–343)	53 ± 28 (10–205)	39 ± 22 (11–181)	49 ± 30 (16–343)
TZ MRI-based	31 ± 26 (0.4–332)	33 ± 26 (0.4–169)	24 ± 19 (2–144)	30 ± 29 (4–332)
PSA density (ng/mL/cc) mean ± SD, range				
Whole gland TRUS-based	0.24 ± 0.39 (0.01–9.37)	0.24 ± 0.31 (0.01–4.49)	0.19 ± 0.15 (0.01–1.25)	0.24 ± 0.60 (0.01–9.37)
Whole gland MRI-based	0.26 ± 0.39 (0.01–7.18)	0.27 ± 0.35 (0.01–5.80)	0.30 ± 0.23 (0.04–2.03)	0.22 ± 0.39 (0.01–7.18)
TZ MRI-based	0.55 ± 0.86 (0.01–12.41)	0.58 ± 0.99 (0.01–12.41)	0.64 ± 0.6 (0.05–4.61)	0.43 ± 0.56 (0.03–8.64)

*TRUS* transrectal ultrasound, *PSA* prostate-specific antigen, *TZ* transition zone

**Fig. 2:**
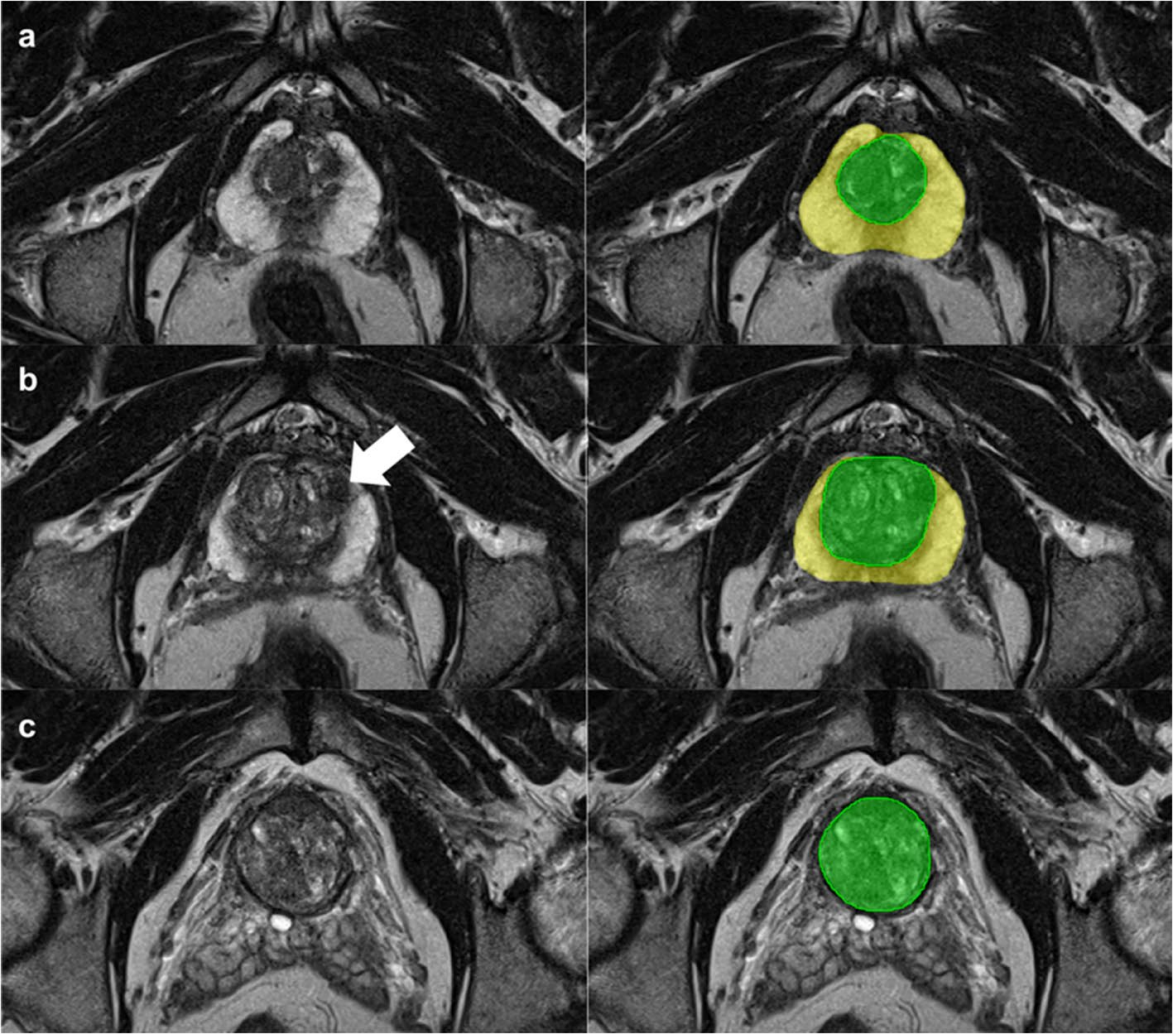
Whole gland and transition zone segmentation at MRI using a nnU-Net. Axial T2-weighted images at three different levels of the prostate (left) and the correlating segmentation masks (right). The yellow and green masks represent the whole gland and transition zone volume, respectively. The white arrow indicates a target biopsy-proven Gleason Score 3+3 prostate cancer in the ventral transition zone

DL-based volume estimates had a segmentation DICE score of 0.89 (95%CI, 0.80–0.97) and 0.84 (0.70–0.99) for the WG and TZ at MRI, respectively, with a mean deviation of 4% from the manually segmented WG volume (2 mL of 50 mL WG; Fig. 2 and Supplementary Figures 1 and 2). The segmentation processing time was 12s (95%CI, 9–15s).

### GG ≥ 2 cancer detection using PSAD alone

**Fig. 3:**
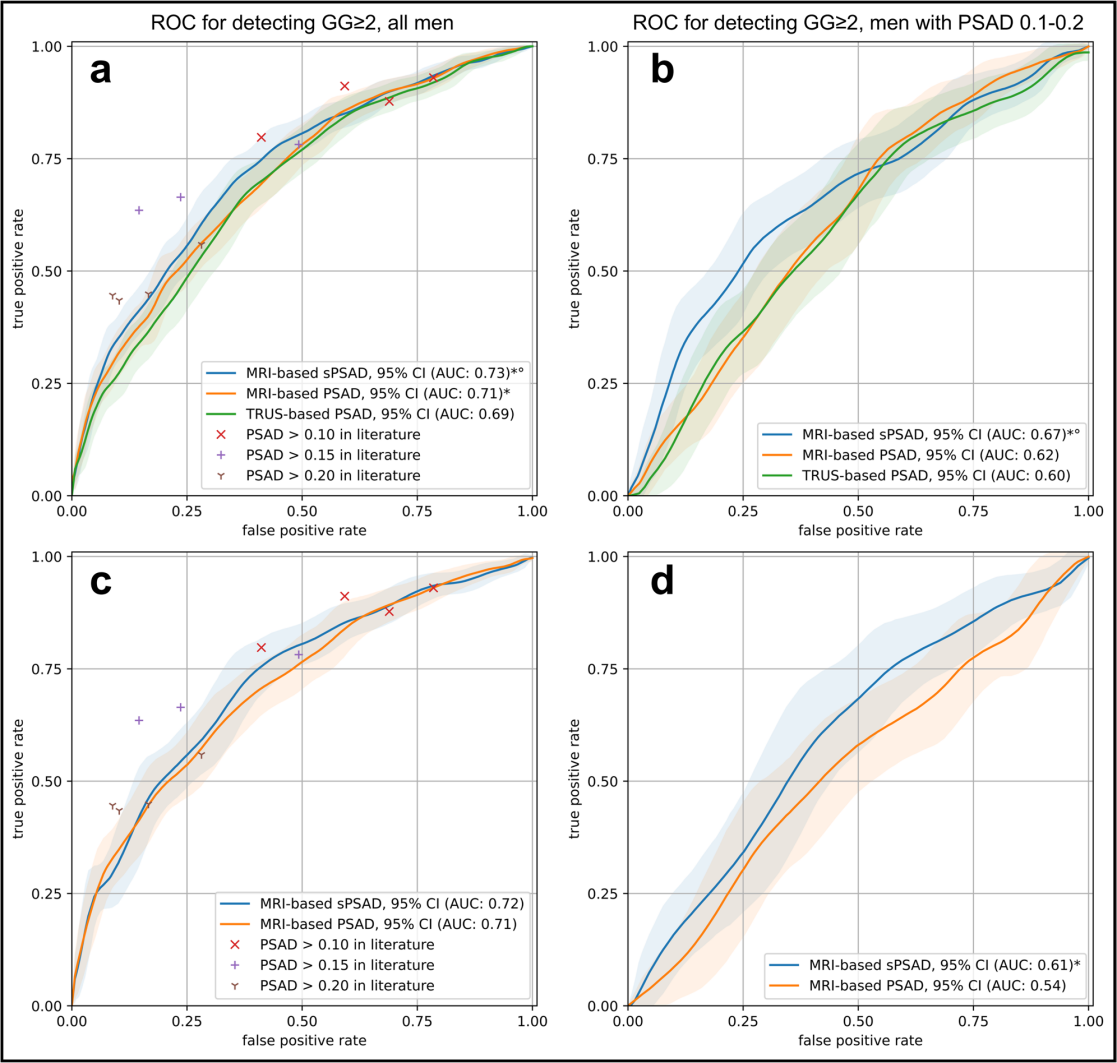
Patient-based diagnostic performance in detecting clinically significant prostate cancer using PSA density in the validation and external test set. **a** Graph shows the receiver operating characteristic (ROC) curves for the detection of ISUP Grade Group (GG) ≥ 2 cancers using transrectal ultrasound (TRUS)- and MRI-based prostate-specific antigen density (PSAD) (green and orange, respectively) and MRI-based transition zone-specific (s)PSAD (blue). Comparator studies with plotted detection accuracy of any-grade PCa and GG ≥ 2 cancers included Boesen et al [11], Knaaplia et al [13], Falagario et al [9], and Hansen et al [8]. Data on cancer detection using a PSAD cutoff of > 0.15 was not available in Hansen et al [8]. **b** Graph shows the ROC curves for the detection of GG ≥ 2 cancers using TRUS- and MRI-based PSAD (green and orange, respectively) and MRI-based sPSAD (blue) in patients with an MRI-based PSAD of 0.1–0.2. **c**, **d** Graph shows the ROC curves for the detection of GG ≥ 2 cancers using MRI-based PSAD and sPSAD in the external test set (*n *= 692) [31]. 95%CIs are shown as transparent areas around the mean curves. 95%CI were estimated through bootstrapping. *Significantly superior performance in comparison to TRUS-based PSAD. °Significantly superior performance in comparison to MRI-based PSAD. (*AUC* area under the ROC curve)

sPSAD achieved an AUC of 0.73 (95%CI, 0.70–0.76) for GG ≥ 2 detection, outperforming TRUS- and MRI-based PSADs (AUC, 0.69 (0.66–0.72) and 0.71 (0.68–0.74); *p* < 0.001; Fig. 3a). The performance of PSAD for GG ≥ 2 and any-grade PCa is comparable to literature that distribute within the CI of the plotted AUCs (Supplementary Figure 3).

In patients with clinically challenging PSAD (intermediate-low to intermediate-high risk; 0.1–0.2 ng/mL/cc; Fig. 3b), TRUS- and MRI-based PSAD for GG ≥ 2 was similar (AUC, 0.60 (95%CI, 0.54–0.66) vs. 0.62 (0.57–0.67); *p* = 0.61) while both were outperformed by the sPSAD (0.66 (0.61–0.71); *p* = 0.031 and *p* = 0.023 in comparison to TRUS- and MRI-based PSAD, respectively). On external testing without additional refinements or retraining, sPSAD performed similarly to PSAD (AUC, 0.72 (0.68–0.76) vs 0.71 (0.67–0.75); *p* = 0.13) but outperformed the whole gland approach in patients with clinically challenging PSAD (0.61 (0.54–0.68) vs 0.54 (0.47–0.61); *p* = 0.018; Fig. 3c–d).

### Biopsy decision strategy using PI-RADS and sPSAD

#### Performance of the sPSAD-based BDS in the validation set

Performing biopsy in men with PI-RADS 3-5 categories achieved a sensitivity of 100% (95%CI, 100–100%) and specificity of 24% (16–32%) for GG ≥ 2 detection. Following the recommended approach of performing biopsy in men with a PI-RADS 4-5 and PI-RADS 3 if the density is above a designated cutoff, the best-performing BDS achieved a sensitivity of 100% (95%CI, 100–100%; *p* = 1) and specificity of 36% (28–47%; *p* = 0.027), utilizing a sPSAD cutoff of 0.42 ng/mL/cc for GG ≥ 2. In comparison, the clinically established BDS using the MRI-based PSAD cutoff of 0.15 ng/mL/cc resulted in a sensitivity of 100% (95%CI, 100–100%; *p* = 1) and specificity of 33% (23–41%; *p* = 0.11) for GG ≥ 2 cancers. Any-grade PCa detection results are provided in the supplementary material.

#### Testing of the sPSAD-based BDS in the hold-out test set

PI-RADS 3-5 achieved a sensitivity and specificity of 97% (95%CI, 94–99%) and 18% (10–20%) for GG ≥ 2, respectively. The developed sPSAD-based BDS achieved a sensitivity of 93% (95%CI, 89–96%; *p* = 0.053) and specificity of 43% (30–44%; *p* < 0.001) for GG ≥ 2, when applying a sPSAD cutoff of 0.42 ng/mL/cc. In comparison, the established BDS with a PSAD cutoff of 0.15 achieved a sensitivity of 93% (95%CI, 89–96%; *p* = 0.053) and a lower specificity of 34% (27–41%; *p* < 0.001).

#### Overall impact on biopsy decision and outcome

**Fig. 4:**
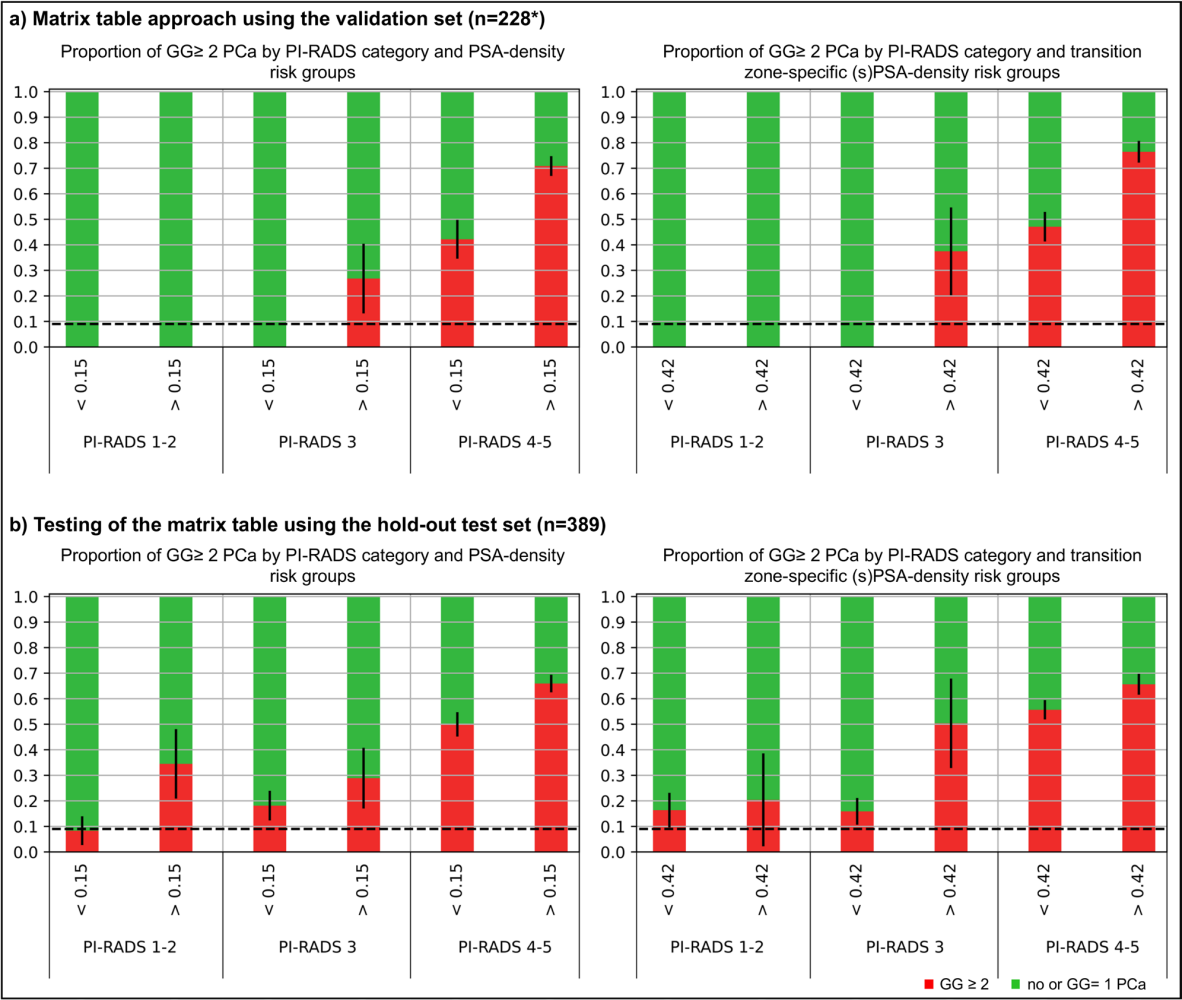
Proportions of clinically significant prostate cancer related to PI-RADS and PSA density risk category in men planned for prostate biopsy. Proportions of clinically significant prostate cancers (ISUP GG ≥ 2; red bars) and GG = 1 or no cancers (green bars), related to PI-RADS score categories (1–2, 3, 4–5) at whole gland PSA densities (PSAD; < 0.15 and ≥ 0.15 ng/mL/cc) as well as transition zone-specific (s)PSAD (< 0.42 and ≥ 0.42 ng/mL/cc) risk categories, respectively, in the validation and hold-out test sets. The overall accepted risk threshold of 9% for GG ≥ 2 by 2019 EAU prostate cancer guidelines, when using prostate MRI for biopsy decisions, is plotted as dashed horizontal line. The diagram illustrates that men without GG ≥ 2 are more likely to have a sPSAD below 0.42 than a PSAD below 0.15. *Fifteen of the 243 men in the validation set had to be excluded from the analysis as no PI-RADS score could be determined

Applying the proposed PI-RADS and sPSAD-based risk groups, men with a PI-RADS 4-5 or PI-RADS 3 with sPSAD ≥ 0.42 ng/mL/cc showed more men with GG ≥ 2 cancers (prevalence of 38–78%; Fig. 4). Based on the overall EAU accepted risk threshold of 9% for GG ≥ 2 [4], a biopsy could have safely been avoided in men with a PI-RADS 3 and a sPSAD of < 0.42 in the validation set. However, a GG ≥ 2 prevalence of 16% (95%CI, 11–21%) was detected for the same risk group in the hold-out test set. Note also that in the hold-out test set, PI-RADS 1/2 demonstrated an intermediate (16–20%) GG ≥ 2 risk in men with a sPSAD < 0.42 and ≥ 0.42.

**Fig. 5:**
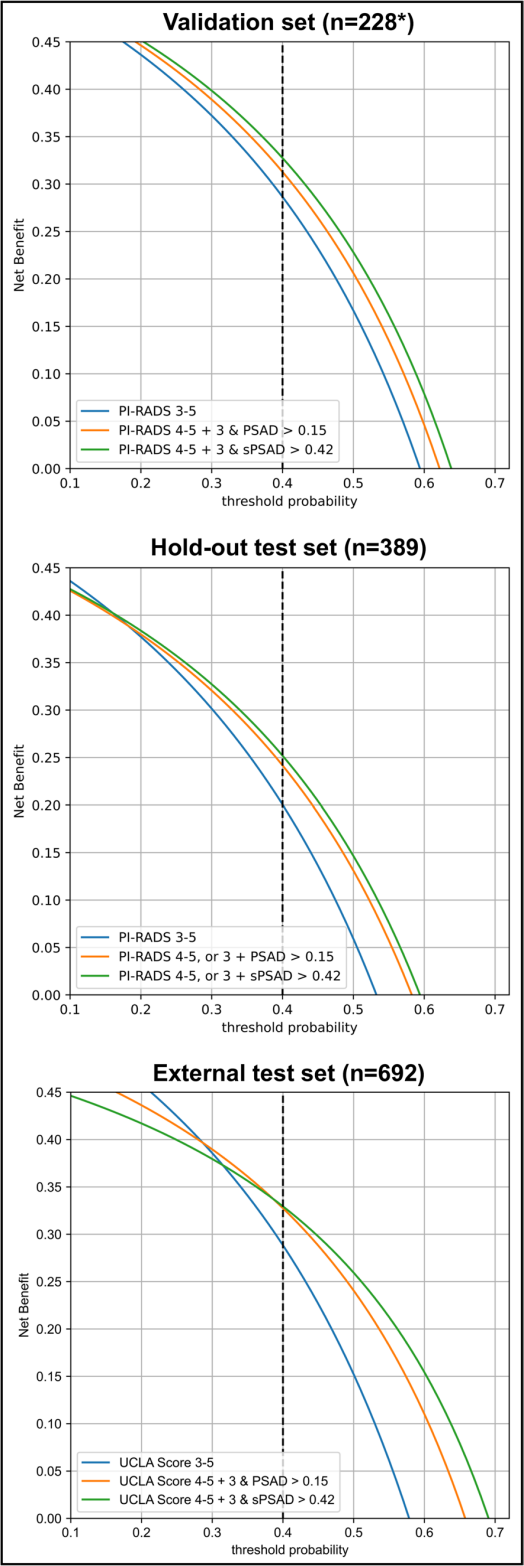
Decision curve analysis comparing clinical utility of different biopsy strategies for detecting clinically significant prostate cancer in men planned for prostate biopsy. Decision curve analyses simulate two scenarios: in one all the men with PI-RADS 3-5 would receive biopsy (PI-RADS 3-5, blue), and in the other none would undergo biopsy (zero on x-axis). Clinically useful biopsy decision strategies lie above these scenarios. The graph gives the expected net benefit per patient relative to biopsy none. The unit is the benefit associated with one patient having GG ≥ 2 duly undergoing biopsy. In internal and external datasets, at a 40% biopsy threshold (=2 out of 5 biopsies yield GG ≥ 2 cancer), the sPSAD-based biopsy decision strategy (BDS) had a net benefit compared to PI-RADS/UCLA 3-5 and PSAD-based BDS. Note how incorporating PSAD improved the net benefit of the MRI strategies in all datasets. *Fifteen of the 243 men in the validation set had to be excluded from the analysis as no PI-RADS score could be determined. (*PI-RADS* Prostate Imaging-Reporting and Data System; *(s)PSAD* (transition zone-specific) prostate-specific antigen density; *UCLA* Likert-like scoring system, similar to PI-RADS v2)

DCA revealed that the BDS using PI-RADS 4/5 or PI-RADS 3 and sPSAD ≥ 0.42 resulted in the highest clinical benefit (Fig. 5), avoiding unnecessary biopsies in 18% (95%CI, 12–23%) and 19% (15–22%), and reducing the false-positive rate from 41% (33–48%) to 36% (29–43%; *p* < 0.001) and 47% (42–52%) to 41% (35–46%; *p* = 0.027) in the validation and hold-out test sets, respectively (Table 3). At the same time, GG ≥ 2 cancer was only missed in 0% (95%CI, 0–0%) and 7% (3–11%) of cases, respectively. Compared to performing biopsy in all men with PI-RADS 3-5, the number of false-positive findings would have been reduced by 17% (95%CI, 9–25%) and 25% (18–33%) in the validation and hold-out test set (68 (53–82) vs. 82 (67–97; *p* = 0.027) and 123 (104–142) vs. 165 (146–186; *p* < 0.001)), respectively. Additionally, the sPSAD-based BDS performance was similar to the PSAD-based BDS in the DCA, minimally reducing the number of false-positive findings (68 (95%CI, 53–82) vs. 73 (58–88); *p* = 0.28) and improving biopsy avoidance (123 (104–142) vs. 129 (111–148; *p* = 0.28) with an identical number of missed GG ≥ 2 cancers. Finally, the use of the developed sPSAD-based BDS in the external set, which substantially differed from our cohort (supplementary material), resulted in the highest clinical benefit compared to MRI scoring alone or in combination with PSAD (Fig. 5).

**Table 3:** Diagnostics metrics for clinically significant prostate cancer detection at various thresholds of PI-RADS score and whole gland and transition zone-specific (s)PSA density risk category, related to biopsy avoidance

Validation set (n = 228*)					
Biopsy strategy	Men biopsied	Biopsies avoided	ISUP GG ≥ 2 cancers		Biopsies without ISUP GG ≥ 2 cancers (FP)
			Detected (TP)	Missed (FN)	
PI-RADS 1-5 (reference)	228	0	120	0	108 (47%)
PI-RADS 3-5	202 (89%)	26 (11%)	120 (100%)	0 (0%)	82 (41%)
PI-RADS 4-5	181 (79%)	47 (21%)	117 (98%)	3 (2%)	64 (35%)
PI-RADS 4-5 + 3 & PSAD ≥ 0.15	193 (85%)	35 (15%)	120 (100%)	0 (0%)	73 (38%)
PI-RADS 4-5 + 3 & sPSAD ≥ 0.42	188 (83%)	40 (18%)	120 (100%)	0 (0%)	68 (36%)
*Hold-out test set (n = 389)*					
Biopsy strategy	Men biopsied	Biopsies avoided	ISUP GG ≥ 2 cancers		Biopsies without ISUP GG ≥ 2 cancers (FP)
			Detected (TP)	Missed (FN)	
PI-RADS 1-5 (reference)	389	0	194	0	195 (50%)
PI-RADS 3-5	353 (91%)	36 (9%)	188 (97%)	6 (3%)	165 (47%)
PI-RADS 4-5	295 (76%)	94 (24%)	176 (91%)	18 (9%)	119 (40%)
PI-RADS 4-5 + 3 & PSAD ≥ 0.15	309 (79%)	80 (21%)	180 (93%)	14 (7%)	129 (42%)
PI-RADS 4-5 + 3 & sPSAD ≥ 0.42	303 (78%)	86 (22%)	180 (93%)	14 (7%)	123 (41%)

Fifteen men were missing a Prostate Imaging-Reporting and Data System (PI-RADS) score in the validation set (*). *ISUP GG* International Society of Urological Pathology Grade Group, *TP* true positive, *FN* false negative, *FP* false positive

## Discussion

PSAD is an independent predictor of GG ≥ 2 cancers and is a valuable asset in BDS combined with PI-RADS scoring [4, 17, 38]. However, a TZ-specific (s)PSAD approach, which specifically accounts for PSA increase due to benign prostatic hyperplasia and larger TZ volume, could be a better predictor of biopsy results. To address this, our study presents an approach using PI-RADS and sPSAD for GG ≥ 2 cancer detection, where TZ volumetry was performed semiautomatically using a DL segmentation tool. sPSAD improved the detection of GG ≥ 2 PCa in an internal and external test set compared to PSAD particularly in men with a grey zone PSAD (PSAD of 0.1–0.2 ng/mL/cc). Our BDS using PI-RADS scoring and sPSAD, which recommended biopsy in men with PI-RADS 4/5 or PI-RADS 3 and sPSAD ≥ 0.42, improved the specificity for GG ≥ 2 detection, while sensitivity was not significantly reduced compared to a BDS of PI-RADS 3-5 only. The developed sPSAD-based BDS was the best-performing strategy for GG ≥ 2 diagnosis in DCA, improving biopsy avoidance and reducing the number of false positives while maintaining high sensitivity compared to preforming biopsy in men with a PI-RADS 3-5 only.

The issue of a high false-positive rate of up to 45% in PI-RADS 3-5 cases has been tackled by developing image-based BDS using PI-RADS scoring and PSAD, aiming to improve the positive predictive value of MRI-guided biopsy. In this context, the EAU guideline recommended prostate biopsy in all men with elevated risk for GG ≥ 2 cancer (prevalence ≥ 30%; with PI-RADS 4-5 or PSAD ≥ 0.2 ng/mL/cc), and biopsy should highly be considered in men with an intermediate risk (≥ 20%; PI-RADS 4-5 or PSAD 0.15–0.2) [17]. These EAU risk groups were confirmed by our study achieving similar yields of GG ≥ 2 disease. However, our results show that a zone-specific approach for PSAD calculation would improve both biopsy avoidance and address the need to reduce false positives on prostate MRI.

The adoption of the proposed sPSAD-based BDS is likely to offer immediate clinical advantages in routine practice. This newly proposed strategy demonstrated the highest clinical yield in a well-balanced cohort comprising both biopsy-naive and biopsy-negative men (validation set), as well as in an MRI-first cohort, where over 80% of the individuals were biopsy-naïve (hold-out test set). Specifically, the employment of sPSAD-based BDS led to a 9% increase in specificity for the detection of GG ≥ 2 PCa, as compared to the BDS using whole gland PSAD, while showing identical sensitivity. Moreover, our approach of using a DL tool for sPSAD calculation proved its feasibility in an external test set and can be easily implemented by minor modifications of currently available software, thus enabling clinical translation of the method.

While the study results are promising, there are limitations. First, clinical implications derived from this single-center investigation may be limited by the retrospective study design. However, this study included a large number of consecutive patients, keeping the risk of a selection bias to a minimum. Moreover, clinical utility and feasibility were tested on an internal and external test set and our results on any-grade PCa and GG ≥ 2 detection using PSAD alone and in the context of PI-RADS-based risk groups were similar to published literature [8, 9, 11, 13]. Second, our analysis is limited by the uncertainty over the prevalence of GG ≥ 2 cancer in the study sample, since men did not undergo saturation biopsy. However, this is a limitation of most PCa diagnosis studies. Templated biopsies are the gold standard in the diagnosis context, but our institution preforms MRI/US fusion-guided targeted and systematic biopsy in men with suspicious lesions at MRI, which is in accordance with the EAU guidelines and the MRI diagnosis pathway [2]. Nonetheless, targeted biopsy was not performed in all patients based on MRI risk estimation. However, if only cases with MRI-targeted biopsy had been included, we would have suffered a selection bias in favor of cases with MRI-positive findings, while MRI-invisible PCa potentially would have been excluded. Third, the retrospective PI-RADS consensus review in this study may have differed from the original report, but cases with distinct radiological-pathological discrepancies were excluded. Thus, the overall impact on diagnostic performance can be considered marginal. Fourth, clinical implications of the sPSAD-based BDS at the accepted risk by the EAU guidelines of missing 10% of csPCa could not be demonstrated as PI-RADS 4/5 alone achieved a sensitivity of > 90% [4]; however, our higher population prevalence of 48% is in line with the results of the PAIREDCAP study [39]. Within these limitations, we demonstrated the initial feasibility of using sPSAD for biopsy decision planning.

In conclusion, our findings underscore the concept and use of a TZ-specific (s)PSAD for image-based prostate biopsy decision planning. sPSAD improved GG ≥ 2 cancer detection when compared to PSAD alone and outperformed PI-RADS only based BDS. Specifically, the implementation of sPSAD-based BDS was able to reduce false positives at MRI and improve biopsy avoidance, while maintaining a high detection rate of clinically significant disease. Thus, the use of sPSAD for biopsy decisions should be evaluated in prospective studies.

## Supplementary Information

Below is the link to the electronic supplementary material.

Supplementary file1 (PDF 742 KB)

## Abbreviations

95%CI: 95% confidence interval
AUC: Area under the ROC
BDS: Biopsy decision strategy
csPCa: Clinically significant prostate cancer
DCA: Decision curve analysis
DL: Deep learning
EAU: European Association of Urology
ISUP GG: International Society of Urological Pathology Grade Group
PI-RADS: Prostate Imaging-Reporting and Data System
PSAD: Prostate-specific antigen density
PV: Prostate volume
ROC: Receiver operating characteristic curve
sPSAD: TZ-specific PSAD
TRUS: Transrectal ultrasound
TZ: Transition zone
WG: Whole gland

## Declarations

### Guarantor

The scientific guarantor of this publication is Tobias Penzkofer.

### Statistics and biometry

Several authors have significant statistical expertise (G.L.B., A.P., T.P.).

### Study subjects or cohorts overlap

A subset of the study population investigated here was previously reported in Hamm et al Radiology 2023 (overlap of 75% (1210 men), development of an explainable deep learning system for prostate cancer detection), Beetz et al 2022 (29%, biopsy accuracy correlated with number of biopsy cores), Rudolph et al European Radiology 2020 (28%, validation of PI-RADS features), Beetz et al RöFo 2022 (20%, intra-reader comparison of PI-RADS version 2 and 2.1), and Rudolph 2020 (6%, intra-reader comparison of PI-RADS version 2 and 2.1). However, zone-specific PSAD and biopsy decision strategies using PSAD and PI-RADS were not investigated for prostate cancer detection.

### Methodology

• retrospective

• diagnostic or prognostic study

• performed at one institution
