# Supplementary material for: Reduction of false positives using zone-specific prostate-specific antigen density for prostate MRI-based biopsy decision strategies
Source: Eur Radiol. 2024 Mar 28;34(10):6229–40. doi: 10.1007/s00330-024-10700-z (PMC11399225; doi:10.1007/s00330-024-10700-z)
Supplement: Supplementary file 1 — Supplementary file1 (PDF 742 KB) [file 330_2024_10700_MOESM1_ESM.pdf]

Reduction of False Positives Using Zone-specific Prostate-Specific Antigen Density for Prostate MRI-based Biopsy Decision Strategies

Electronic Supplementary Material (ESM)

Eur Radiol (2024) Hamm CA, Baumgärtner GL, Padhani AR et al.

Methods

External test set

For external testing the “Prostate MRI and Ultrasound With Pathology and Coordinates of Tracked Biopsy (Prostate-MRI-US-Biopsy)” dataset was used [1]; accessible at https://wiki.cancerimagingarchive.net/pages/viewpage.action?pageId=68550661.

MRIs were not reported using the PI-RADS scoring system but were scored using the Likert-like "UCLA score”, which is similar to PI-RADS v2. Of 1151 patients, 309 and 4 men were excluded due to missing PSA values or MRI scan. Further, 146 patients had multiple MRI examinations and were therefore excluded from analysis. Finally, 692 men were included in the final analysis with a GG≥2 cancer prevalence of 58%.

UCLA distribution:

UCLA score	No. of patients
1	17
2	4
3	241
4	240
5	190

GG≥2 cancer distribution according to UCLA:

UCLA score	No. of patients with GG≥2 cancer
1	9
2	1
3	91
4	130
5	168

Deep learning segmentation model

The nnU-Net version 2.0 [2] was used unchanged. The model consists of an 3-D and 2-D U-Net [2] architecture and is trained in a 5-fold cross-validation approach for 1000 epochs with custom data augmentation [3] including for example scaling from 70% to 140%, gaussian noise, and simulated low resolution.

Segmentation performance assessment

In addition to the DICE coefficient, we evaluated the pairwise agreement between PVs derived from DL-based segmentation at MRI, manual segmentation at MRI, ellipsoid formula-based

calculations at MRI, and TRUS using the paired t-test and Bland-Altman plots (Supplementary Figure 1).

Results

Biopsy decision strategy using PI-RADS and sPSAD for any-grade PCa detection

Performing biopsy in men with PI-RADS 3-5 categories achieved a sensitivity of 96% (95% confidence interval (95%CI): 94-98%) and specificity of 25% (17-34%) for any-grade PCa detection in the validation set. Applying the developed sPSAD-based BDS, following the recommended approach of performing biopsy in men with a PI-RADS 4-5 and PI-RADS 3 if the density is above a designated cutoff, the best-performing BDS utilized a sPSAD cutoff of 0.2 for PCa with a sensitivity of 94% (95%CI: 91-97%; p=0.126, when compared to PI-RADS 3-5 only) and specificity of 46% (36-56%; p=0.001).

In the hold-out test set, PI-RADS 3-5 achieved an sensitivity and specificity of 98% (95%CI: 96-100%) and 48% (33-62%) for any-grade PCa, respectively. The developed sPSAD-based BDS using a sPSAD cutoff of 0.2 achieved a sensitivity of 98% (95%CI: 96-100%p=0.12) and specificity of 60% (45-75%; p<0.001) for any-grade PCa.

Using UCLA and sPSAD for csPCa detection in an external test set

Using the trained nnU-Net without additional refinements or retraining for prostate segmentation, the mean volume deviation from the segmented reference volume was 0.6%.

UCLA 3-5 achieved a sensitivity and specificity of 98% (95%CI: 96-99%) and 4% (2-6%) for GG≥2, respectively. The developed sPSAD-based BDS achieved a sensitivity of 82% (95%CI: 78-85%; p<0.001) and specificity of 50% (45-56%; p<0.001) for GG≥2, when applying a sPSAD cutoff of 0.42 ng/mL/cc. In comparison, the established BDS with a PSAD cutoff of 0.15 achieved a sensitivity of 87% (95%CI: 84-91; p<0.001) and lower specificity of 39% (95%CI: 33-44%; p<0.001). In men with clinically challenging PSAD (intermediate-low to intermediate-high risk; 0.1-0.2 ng/mL/cc), sPSAD-based BDS increased specificity significantly

(42% (95%CI: 35-49%) vs. 30% (23-37%); p=0.032) while no significant loss in sensitivity was shown compared to a PSAD-based BDS utilizing a cutoff of 0.15 (77% (70-84%) vs. 84% (77-91%); p=0.12; **Supplementary Table 1**). Moreover, DCA revealed that the sPSAD-based BDS resulted in the highest clinical benefit in all patients and those with clinically challenging PSAD (**Supplementary Figure 4**).

Supplementary Table 1: Diagnostics metrics for clinically significant prostate cancer detection at various thresholds of UCLA score and whole gland and transition zone-specific (s)PSA-density risk category in men with clinically challenging PSA-density

Biopsy strategy	men biopsied	biopsies avoided	ISUP GG≥ 2 cancers		biopsies without ISUP GG≥ 2 cancers (FP)
			detected (TP)	missed (FN)	
**UCLA 1-5 (reference)**	271	0	146	0	125 (46%)
**UCLA 3-5**	261 (96%)	10 (4%)	143 (98%)	3 (2%)	125 (45%)
**UCLA 4-5**	167 (62%)	104 (38%)	104 (71%)	42 (29%)	63 (38%)
**UCLA 4-5 + 3 & PSAD ≥ 0.15**	210 (78%)	61 (23%)	122 (84%)	24 (16%)	88 (42%)
**UCLA 4-5 + 3 & sPSAD ≥ 0.42**	186 (69%)	85 (31%)	113 (77%)	33 (23%)	73 (39%)

Note.- ISUP GG= International Society of Urological Pathology Grade Group, TP= true positive, FN= false negative, FP= false positive.

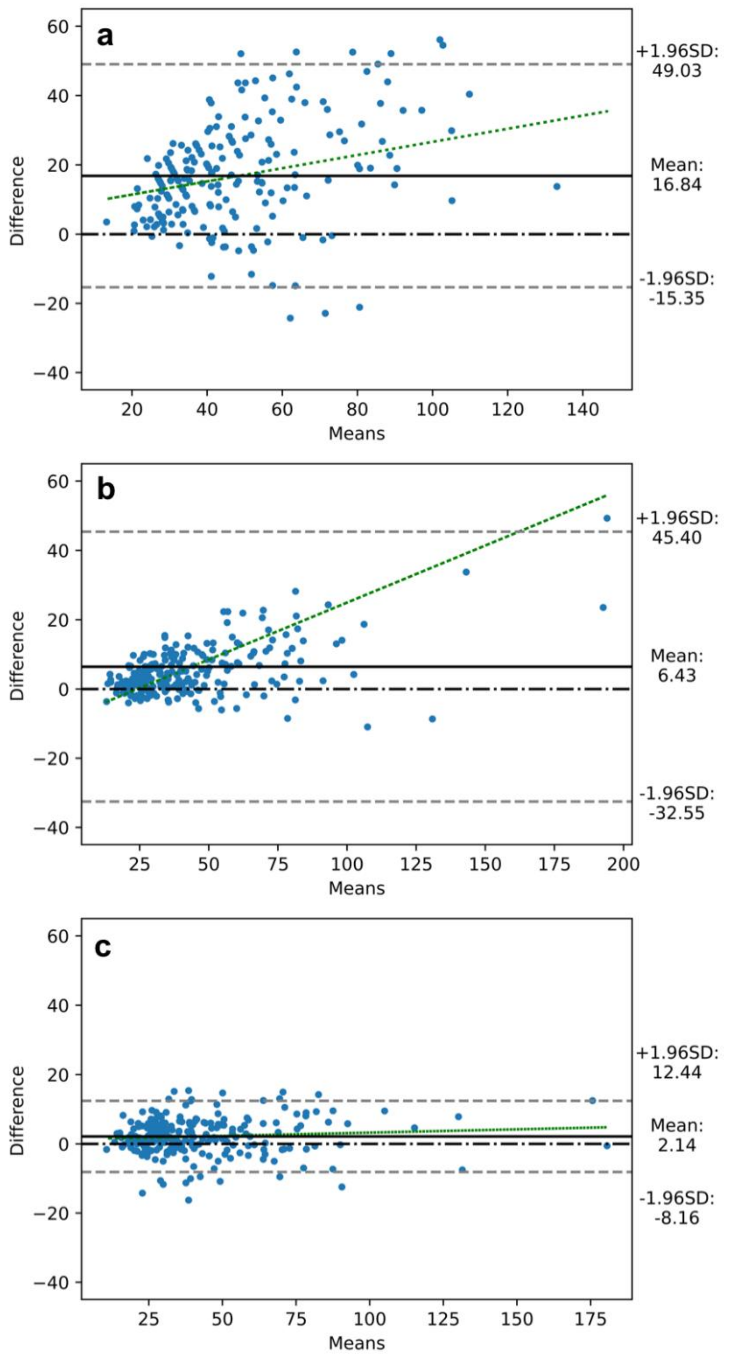

Supplementary Figure 1: Bland-Altman plot demonstrating the agreement of prostate volumetry at MRI and transrectal ultrasound.

Graph (**a**) demonstrates the agreement of prostate volumetry using the ellipsoid formula on transrectal ultrasound and manual prostate segmentations at MRI. Graph (**b**) demonstrates the agreement of prostate volumetry using the ellipsoid formula and manual prostate segmentations at MRI. Graph (**c**) demonstrates the agreement of manual and deep learning-based semi-automated segmentations at MRI. Data plotted here stem from the validation set,

comprising 243 men. The identified (dash-dotted line), mean difference (solid line), 95%CI of limits of agreement (dashed lines), and regression lines (green dotted line) are plotted. A high variability between the volume estimates between TRUS and MRI can be seen with a +17 ml (95%CI: -15 to +49 ml) bias towards ultrasound (**a**). Variability decreases when PV estimation is performed at MRI using the ellipsoid formula with a +6 ml (95%CI: -33 to +45 ml) bias towards the ellipsoid-based PV (**b**). The agreement between the manual and semiautomated MRI-based segmentation methods (**c**) has a significantly lower bias (p<0.001) and variability with +2 ml (95%CI: -8 to +12 ml) compared to **a** and **b**.

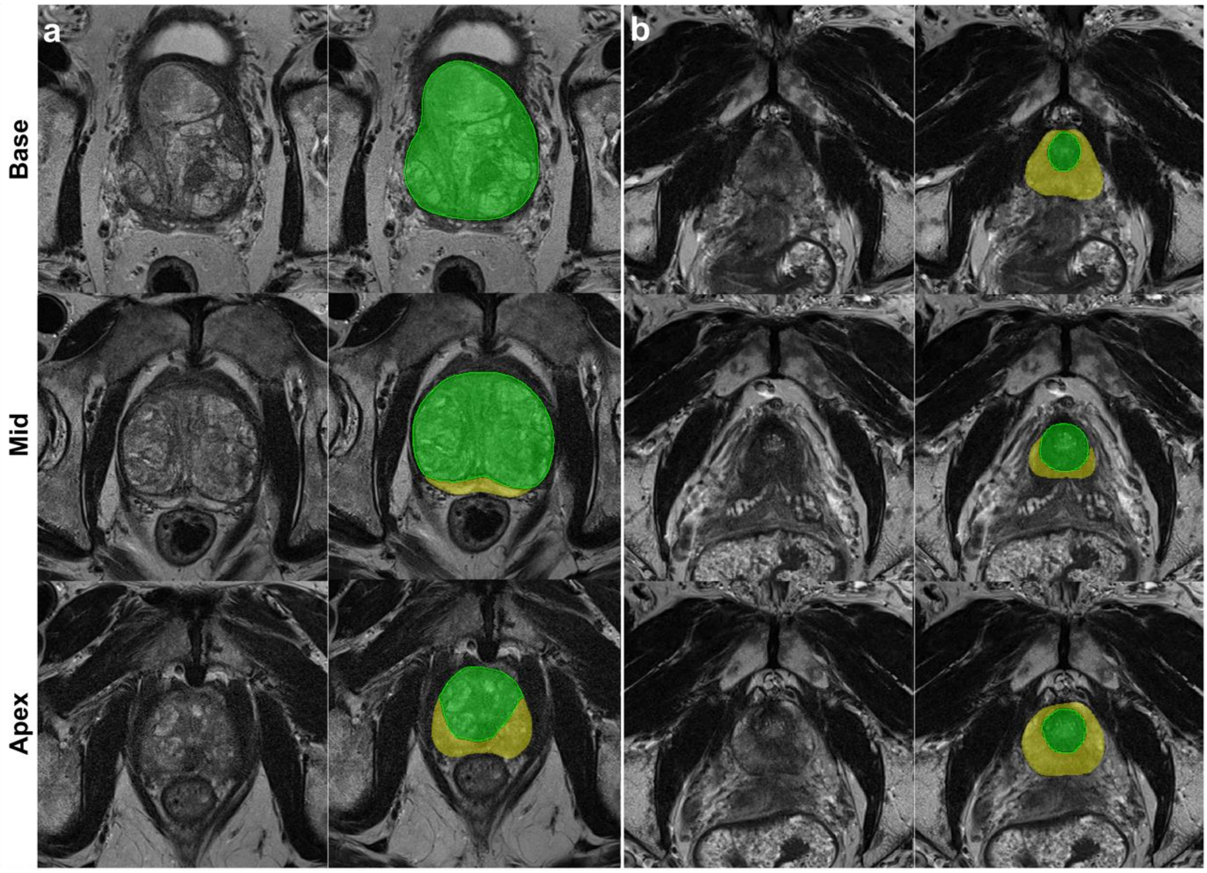

Supplementary Figure 2: Whole gland and transition zone segmentation of very large and very small prostates at MRI using a nnU-Net.

Axial T2-weighted images at three different levels of the prostate (left) and the correlating segmentation masks (right). The yellow and green masks represents the whole gland and transition zone volume, respectively. On the left (**a**), the prostate of a 70-year-old cancer-free

man with a PSA of 13.75 ng/ml and a prostate volume of 169 ml is segmented with a DICE coefficient of 0.95 for the whole gland and the transitional zone. On the right (**b**), the prostate of a 75-year-old man with GG5 cancer, a PSA of 8.58 ng/ml and a prostate volume of 16 ml is segmented with a DICE coefficient of 0.88 and 0.80 for the whole gland and the transitional zone, respectively.

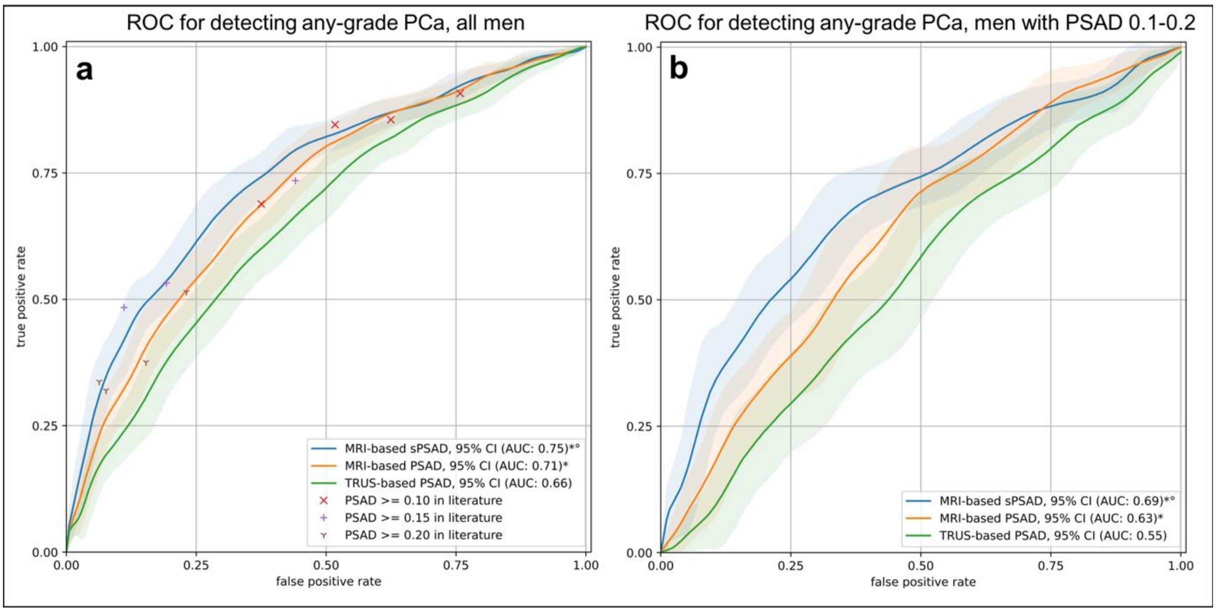

Supplementary Figure 3: Patient-Based diagnostic performance in detecting prostate cancer using PSA-density in the validation set.

(a) Graph shows the receiver operating characteristic (ROC) curves for the detection of any-grade prostate cancer (PCa; Gleason Score ≥6) using transrectal ultrasound (TRUS)- and MRI-based prostate specific antigen density (PSAD) (green and orange, respectively) and MRI-based transition zone-specific (s)PSAD (blue). 95% CIs are shown as transparent areas around the mean curves. Comparator studies with plotted detection accuracy of any-grade PCa and GG≥2 cancers included Boesen et al. 2019 [4], Knaaplia et al. 2019 [5], Falagario et al. 2019 [6], and Hansen et al. 2018 [7]. Data on cancer detection using a PSAD cutoff of >0.15 was not available in Hansen et al. 2018.

(b) Graph shows the ROC curves for the detection of any-grade PCa using TRUS- and MRI-based PSAD (green and orange, respectively) and MRI-based sPSAD (blue) in patients with

an MRI-based PSAD of 0.1-0.2. 95% CIs are shown as transparent areas around the mean curves. AUC= area under the ROC curve. 95% CI were estimated through bootstrapping. *= significantly superior performance in comparison to TRUS-based PSAD. °= significantly superior performance in comparison to MRI-based PSAD.

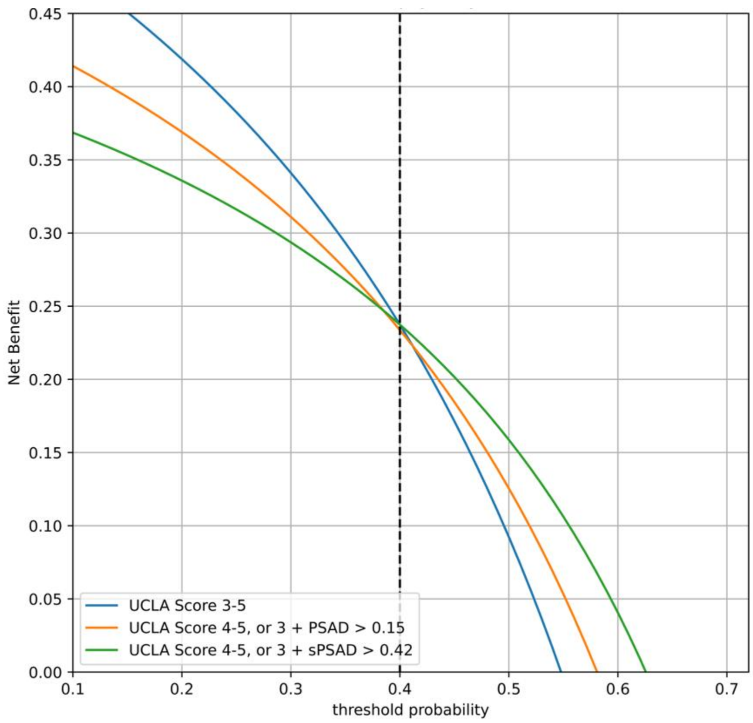

Supplementary Figure 4: Decision curve analysis comparing clinical utility of different biopsy strategies for detecting clinically significant prostate cancer in men with clinically challenging PSA-density.

Decision curve analyses simulate two scenarios: in one all the men with UCLA 3-5 would receive biopsy (UCLA 3-5, blue), and in the other none would undergo biopsy (zero on x-axis). Clinically useful biopsy decision strategies lie above these scenarios. The graph gives the expected net benefit per patient relative to biopsy none. The unit is the benefit associated with one patient having GG≥2 duly undergoing biopsy. At a 40% biopsy threshold (=2 out of 5 biopsies yield GG≥2 cancer), the sPSAD-based biopsy decision strategy (BDS) had a net benefit compared to UCLA 3-5 and PSAD-based BDS. UCLA = Likert-like scoring system; similar to Prostate Imaging Reporting and Data System v2; (s)PSAD = (transition zone-specific) prostate-specific antigen density.

References:

1 Natarajan S, Priester A, Margolis D, Huang J, Marks L (2020) Prostate MRI and ultrasound with pathology and coordinates of tracked biopsy (prostate-MRI-US-biopsy). Cancer Imaging Arch 10:7937

2 Isensee F, Petersen J, Klein A et al (2018) nnU-Net: Self-adapting Framework for U-Net-Based Medical Image Segmentation. CoRR abs/1809.10486

3 Isensee F, Jäger P, Wasserthal J et al (2020) batchgenerators - a python framework for data augmentation. Zenodo

4 Boesen L, Nørgaard N, Løgager V et al (2019) Prebiopsy biparametric magnetic resonance imaging combined with prostate-specific antigen density in detecting and ruling out Gleason 7–10 prostate cancer in biopsy-naïve men. European urology oncology 2:311-319

5 Knaapila J, Jambor I, Perez IM et al (2020) Prebiopsy IMPROD biparametric magnetic resonance imaging combined with prostate-specific antigen density in the diagnosis of prostate cancer: an external validation study. European urology oncology 3:648-656

6 Falagario UG, Martini A, Wajswol E et al (2020) Avoiding unnecessary magnetic resonance imaging (MRI) and biopsies: negative and positive predictive value of MRI according to prostate-specific antigen density, 4Kscore and risk calculators. European urology oncology 3:700-704

7 Hansen NL, Barrett T, Kesch C et al (2018) Multicentre evaluation of magnetic resonance imaging supported transperineal prostate biopsy in biopsy-naïve men with suspicion of prostate cancer. BJU Int 122:40-49
